# Functional network integration and attention skills in young children

**DOI:** 10.1016/j.dcn.2018.03.007

**Published:** 2018-03-20

**Authors:** Christiane S. Rohr, Anish Arora, Ivy Y.K. Cho, Prayash Katlariwala, Dennis Dimond, Deborah Dewey, Signe Bray

**Affiliations:** a Department of Radiology, Cumming School of Medicine, University of Calgary, Calgary, Alberta, Canada; b Department of Paediatrics, Cumming School of Medicine, University of Calgary, Calgary, Alberta, Canada; c Department of Neuroscience, Cumming School of Medicine, University of Calgary, Calgary, Alberta, Canada; d Department of Community Health Sciences, University of Calgary, Calgary, Alberta, Canada; e Child and Adolescent Imaging Research Program, University of Calgary, Calgary, Alberta, Canada; f Alberta Children’s Hospital Research Institute, University of Calgary, Calgary, Alberta, Canada; g Hotchkiss Brain Institute, University of Calgary, Calgary, Alberta, Canada

**Keywords:** Attention, Children, Neural networks, Early childhood, Functional connectivity, ICA

## Abstract

Children acquire attention skills rapidly during early childhood as their brains undergo vast neural development. Attention is well studied in the adult brain, yet due to the challenges associated with scanning young children, investigations in early childhood are sparse. Here, we examined the relationship between age, attention and functional connectivity (FC) during passive viewing in multiple intrinsic connectivity networks (ICNs) in 60 typically developing girls between 4 and 7 years whose sustained, selective and executive attention skills were assessed. Visual, auditory, sensorimotor, default mode (DMN), dorsal attention (DAN), ventral attention (VAN), salience, and frontoparietal ICNs were identified via Independent Component Analysis and subjected to a dual regression. Individual spatial maps were regressed against age and attention skills, controlling for age. All ICNs except the VAN showed regions of increasing FC with age. Attention skills were associated with FC in distinct networks after controlling for age: selective attention positively related to FC in the DAN; sustained attention positively related to FC in visual and auditory ICNs; and executive attention positively related to FC in the DMN and visual ICN. These findings suggest distributed network integration across this age range and highlight how multiple ICNs contribute to attention skills in early childhood.

## 1 Introduction

Early childhood is a particularly crucial period in a child’s development when many cognitive skills, including top-down attention, are rapidly maturing (Brown and Jernigan, 2012). For many children, this is the beginning of formal reading instruction and attention skills appear to be foundational for reading acquisition (Franceschini et al., 2012). More generally, as children approach school age they are increasingly expected to attend to arbitrary symbols, such as numbers and letters (Sørensen and Kyllingsbaek, 2012). Thus, the pre- and early-school period represents a time when attentional demands placed on children not only increase, but also expand to include symbolic stimuli that require considerable perceptual expertise (Ristic and Enns, 2015). Relatively weak attention skills can place children at a disadvantage in school, which can have lifelong consequences on academic attainment, employment, and social skills (Rueda et al., 2010; Stevens and Bavelier, 2012). However, we have a limited understanding of the neural basis of inter-individual variability in early childhood attention skills.

Top-down, or deliberate, attention can be categorized into three component processes: (1) “selective attention” or “orienting” which refers to the ability to search for an object amongst other similar ‘distracter’ objects, (2) “sustained attention” or “alerting”, the ability to maintain attention for longer periods of time, and (3) “executive attention” or “executive control”, the ability to override pre-potent responses (Breckenridge et al., 2013; Petersen and Posner, 2012). Top-down attention skills show vast changes as children develop and are faced with differing demands (Breckenridge et al., 2013). These changes are associated with unique maturational trajectories (Dye and Bavelier, 2010; Hommel et al., 2004; Lobaugh et al., 1998; Zhan et al., 2011), with the most protracted changes occurring in executive attention (Zhan et al., 2011). These divergent cognitive developmental trajectories suggest that distinct attention components have at least partially distinct neural substrates.

Indeed, it has been proposed the three components of top-down attention may be facilitated by distinct brain networks (Petersen and Posner, 2012; Posner and Petersen, 1990). The top-down “orienting network” is centered on the intraparietal sulcus (IPS) and the frontal eye fields (FEF), and is otherwise known as the dorsal attention network (DAN) (Casey et al., 2005; Rohr et al., 2017). In adults, functional connectivity (FC) between the IPS and FEF is enhanced during selective attention (Szczepanski et al., 2013), and FC between the IPS and visual regions is increased during sustained visual attention (Greenberg et al., 2012; Lauritzen et al., 2009). The “alerting network” and “executive network” largely overlap; both contain the IPS as well as the dorsolateral prefrontal cortex (DLPFC), anterior cingulate cortex (ACC), anterior insula, and thalamus, and as such combine features of the frontoparietal (FPN) and cingulo-opercular networks (Petersen and Posner, 2012; Xuan et al., 2016). In adults, sustained and executive attention skills have been associated with FC of FPN nodes at rest (Markett et al., 2014).

Although data from adults suggests associations between functional network organization and attentional skills, few studies to date have linked intrinsic connectivity networks (ICNs) to specific attentional abilities in childhood. In infants and toddlers, significant positive associations between FC and joint attention were observed between the visual network, the DAN and the default mode network (DMN) (Innocenti and Price, 2005; Thompson et al., 2000). In children aged 7–9 years, an association between the synchronization of primary auditory network and attention skills was shown (Seither-Preisler et al., 2014). Children aged 10–14 were found to show greater functional activity in the anterior cingulate, precentral gyrus, amygdala and fusiform gyrus in relation to selective and executive attention (Cascio et al., 2007; Lebel et al., 2008). Data in early childhood are particularly limited. We previously investigated the link between attention and functional brain development in the dorsal attention network (DAN) using a hypothesis-driven seed-based FC approach (Rohr et al., 2017). In line with the established role of the DAN in attention (Corbetta and Shulman, 2002), we observed that stronger selective attention skills predicted greater connectivity strength between DAN nodes with increasing age (Rohr et al., 2017). However, in line with the studies on attention in childhood, recent work has shown that sustained attention in both children and adults relies on a multitude of regions outside the canonical attention networks, providing further evidence the DAN is not the only ICN involved in top-down attention processes (Rosenberg et al., 2016). It is therefore of interest to ask whether distributed functional networks are associated with attention skills in early childhood. In the present study, we therefore examined multiple well-known ICNs in relation to attention skills using Independent Component Analysis (ICA). Specifically, we examined the ICNs extracted from functional magnetic resonance imaging (fMRI) data, which was collected during a passive viewing paradigm in children aged 4–7 years who also completed assessments of sustained, selective and executive attention skills.

We reasoned that given the substantial positive association between age and attention skills in this age range, networks that are associated with attention are likely to be those that show functional integration with age. We therefore began by examining associations between age and FC in our sample. Although the literature shows that the functional networks found in adults are present in children, there appear to be ongoing changes in the degree of “integration” of some regions into functional brain networks (greater intra-network FC) and “segregation” of sets of regions into separate functional networks (less inter-network FC) as children get older (Gu et al., 2015; Kaufmann et al., 2017; Marek et al., 2015; Menon, 2013; Power et al., 2010) (for a comprehensive developmental review see (Grayson and Fair, 2017)). Few studies have specifically addressed changes in early childhood. In children aged 5–8 years, developing ICNs were found to be more diffuse and fragmented compared to adults (de Bie et al., 2012). Localized associations with local and global activity and connectivity measures were recently shown in 2–6 year old children (Long et al., 2017), with prominent age effects in dorsal frontal and parietal regions. Studies extending into later childhood and adolescence also suggest functionally dependent ICN maturation. For example, sensory ICNs (e.g. visual) appear to show a linear trajectory of integration from childhood to adulthood, whereas the maturation trajectory of cognitive ICNs appears to be non-linear, with integration followed by segregation phases sometime in adolescence (Jolles et al., 2011), and heterogeneity across networks (Muetzel et al., 2016). However, the details of ICN maturational trajectories are still being worked out (Grayson and Fair, 2017) and more studies are needed, particularly in early childhood.

Here we present findings using dual-regression ICA, a technique with high test-retest reliability (Chen et al., 2015; Zuo et al., 2010; Zuo and Xing, 2014), to assess associations between ICNs and age. We then report associations between FC in these ICNs and attention skills after controlling for age. We hypothesized that cognitive, as well as sensory, ICNs would show functional integration across this period, and that greater integration in these ICNs would be associated with greater sustained, selective and executive attention skills.

## 2 Methods

### 2.1 Participants

Eighty typically developing (TD) female children between the ages of 4 and 7 years were recruited to participate in this study as part of an ongoing study of genetic disorders affecting girls. This study was approved by the Conjoint Health Research Ethics Board at the University of Calgary and conducted at the Alberta Children’s Hospital. Informed consent was obtained from the parents and informed assent from participating children. Potential participants were excluded if they had a history of neurodevelopmental or psychiatric disorders, neurological problems, were born earlier than 37 weeks gestation or had other medical problems that prevented participation. Participants’ data were evaluated for outliers in behavioral scores and motion on the fMRI scans. For the behavioral measures, outliers were defined as >3 SD from the mean. No participants were excluded because of outlier scores. A total of twenty participants were excluded: 3 were unable to successfully complete the practice session in the MR simulator, 15 had excessive head motion on their fMRI scan (as described below), one fell asleep during fMRI acquisition, and one received an exclusionary diagnosis within 12 months of data acquisition. The final sample consisted of 60 participants (mean age = 5.54 ± 0.79 SD years; IQ range 90–137; mean = 110.7 ± 9.7 SD). Six of these participants were described as non right-handed by their parents: 3 were described as left-handed, 3 as more left-handed than right-handed, and 1 was described as ambidextrous. Handedness was therefore included as a regressor of no interest in the fMRI analysis.

### 2.2 Data acquisition: procedure

Cognitive assessments and MR imaging were collected over two separate two-hour sessions that took place within two weeks of each other. The first session included a general cognitive assessment using the Wechsler Preschool and Primary Scale of Intelligence – 4th Edition^CDN^ (WPPSI-IV ^CDN^) (Wechsler, 2012), a first set of attention measures, and training in an MRI simulator to acquaint the children with the MR environment. During training in the simulator, children watched the same 18-min video that was played during the actual scan and practiced lying still while the sounds of MR scanning were played to them via headphones. If children were not comfortable in the simulator or not able to hold still, data collection was terminated; three children were excluded at this stage. The second visit consisted of the actual MR scanning and children completed the remainder of the attention measures. Attention measures were randomly ordered both across and within data collection days and were conducted in a testing room adjacent to the MR simulator.

### 2.3 Assessment of attention skills

The behavioral assessment consisted of four tasks adapted from the Early Childhood Attention Battery (ECAB) (Breckenridge et al., 2013), a measure designed to reflect the structure of the Test of Everyday Attention for Children (TEA-Ch) (Manly et al., 2001) but appropriate for children 3–6 years of age. Children completed eight sub-tests. Those included in the analyses reported here were measures of visual sustained attention, auditory sustained attention, selective attention and executive attention. All of the subtests, except Visual Search, were administered via a Dell laptop computer (screen size 31 cm by 17.5 cm), at a 35–50 cm viewing distance; auditory items were played through a set of external speakers. All computerized tasks included an initial practice trial, which was repeated if necessary in order for the child to demonstrate that they understood the instructions.

#### 2.3.1 Sustained attention tasks

In the visual sustained attention task, a continuous stream of pictures was presented on a computer screen (200 ms presentations with an inter-stimulus interval (ISI) of 1800 ms) and the child was asked to say “yes“, “animal“, or the name of the animal, when an animal (target) appeared. 30 targets and 120 non-targets (familiar everyday items) were presented and the child received a prompt to pay attention if they missed four consecutive targets. The score was calculated as the number of correct responses minus any errors and prompts. The auditory sustained attention task was carried out and scored in the same way as the visual sustained attention task: here, a continuous stream of words (mono-syllabic animal target words and familiar item non-targets) was presented (average duration 650 ms, ISI 1350 ms) and the child was also asked to say “yes“, “animal“, or the name of the animal, when an animal name was presented. The sustained attention score was then computed as an average between the visual and the auditory sustained attention scores.

#### 2.3.2 Selective attention task

The selective attention task was a visual search task where children were given 60 s to point to targets (18 red apples) among distracters (162 white apples and red strawberries) on a laminated letter-sized search sheet. The experimenter marked items with an erasable marker as children pointed to them. The score was the total number of correctly identified targets.

#### 2.3.3 Executive attention task

The executive attention task was a version of the Wisconsin Card Sorting test (Robinson et al., 1980), adapted for use with young children. Children had to work out which kind of balloon their new teddy bear friend liked. Each trial showed two balloons that varied in color and shape. In stage one, teddy liked balloons of a particular color; in stage two he liked balloons of a different color; and in stage three he liked balloons of a particular shape. Children received feedback on whether their choice was correct, but no other information was given. A total of 20 possible trials were given for each stage, with six consecutive correct responses required for a pass. If a child failed a stage, the test was discontinued. The task was scored as the total number of stages (1, 2, 3) that were successfully completed.

### 2.4 Behavioral analysis

To assess the relationship between age and attention skills, IQ and handedness, Pearson correlations were computed using SPSS 22 (Chicago, IL). As correlations between head motion and behavioral variables of interest can impact FC results (Power et al., 2015), we assessed correlations between all variables and participants’ motion in the scanner: no significant associations were found. Data were checked for outliers (>3 SD from the mean). One outlier was detected in the (auditory or visual) sustained attention assessment; two additional participants were unable to complete the (auditory or visual) sustained attention assessment. For these three participants, a single sustained attention assessment (auditory or visual) was used to calculate the average sustained attention score.

### 2.5 Functional connectivity

#### 2.5.1 Data acquisition

Data were acquired on a 3T GE MR750 w (Waukesha, WI) scanner using a 32-channel head coil. Functional images were acquired in 34 axial slices using a gradient-echo EPI sequence (437 vols, TR = 2500 ms, TE = 30 ms, FA = 70, matrix size 64 × 64, voxel size 3.5 × 3.5 × 3.5 mm^3^; duration: ∼18 min). Children watched a series of clips from “Elmo’s World” inside the MRI for the duration of the functional scan. Passive viewing tasks have previously been used to assess functional activation and connectivity and may provide more stable functional connectivity estimates, with less head motion, than traditional resting tasks (see e.g. (Cantlon and Li, 2013; Vanderwal et al., 2017; Vanderwal et al., 2015; Wang et al., 2017)). Wakefulness was monitored using an SR-Research EyeLink 1000 (Ottawa, ON) infrared camera; only one participant fell asleep during the scan and was excluded from analysis. Anatomical scans were acquired using a T1-weighted 3D BRAVO sequence (TR = 6.764 ms, TE = 2.908 ms, FA = 10, voxel size 0.5 × 0.5 × 0.8 mm^3^).

#### 2.5.2 fMRI data preprocessing

Data preprocessing was done using FSL’s FEAT (Smith et al., 2004). The pipeline included slice-time correction, motion correction, minimal filtering (2000 ms) and spatial smoothing (4 mm Gaussian kernel full-width at half-maximum) prior to denoising. Procedures to mitigate head motion were next performed and are described in more detail in the next section. Finally, the data were normalized to the McConnell Brain Imaging Center NIHPD asymmetrical (natural) pediatric template optimized for ages 4.5–8.5 years (Fonov et al., 2011) followed by normalization to 2 × 2 × 2 mm MNI152 standard space.

#### 2.5.3 Head-motion mitigation procedure

We used a two-step process to address motion confounds in the data. First, we used motion estimates derived from the preprocessing in order to exclude participants with excessive head motion. FD was determined with FSLMotionOutliers, which uses weighted scaling as in (Power et al., 2012) and outputs a list of ‘motion-corrupted’ volumes. These were counted and multiplied by scan TR to establish the amount of scan time affected by motion. Scans were excluded if >8 min had a framewise displacement (FD) of >0.3 mm or >8 mm maximum absolute displacement. Second, we used ICA-based methods to mitigate the impact of head motion on the participants who were retained for analysis, following a recently described procedure (Kaufmann et al., 2017). This approach was chosen because framewise censoring impacts the autocorrelation structure of the data and reduces temporal degrees of freedom (Carp, 2013; Pruim et al., 2015; Yan et al., 2013). Instead, images were denoised using two sets of classifiers trained to distinguish between ‘real’ neural signals and nuisance signals such as motion-induced noise and signal from the white matter and cerebral spinal fluid. Specifically, we used AROMA (running on Python) (Pruim et al., 2015) and FIX (running on FSL/R/MATLAB) (Salimi-Khorshidi et al., 2014), choosing conservative thresholds (‘aggressive’/’20′) in order to decrease the chance of false positives. In recent work, including both FIX and AROMA was shown to be more effective in increasing the temporal signal-to-noise ratio (TSNR) than employing either procedure on their own (Kaufmann et al., 2017). AROMA is an automated procedure that uses a small but robust set of theoretically motivated temporal and spatial features (timeseries and power spectrum) to identify motion artifacts, while FIX was hand-trained on 20 participants, and the resulting classifier was then used to identify noise components in the remaining 40 participants. Noise components identified by AROMA and FIX were then compiled and jointly used to clean the data.

**Fig. 1:**
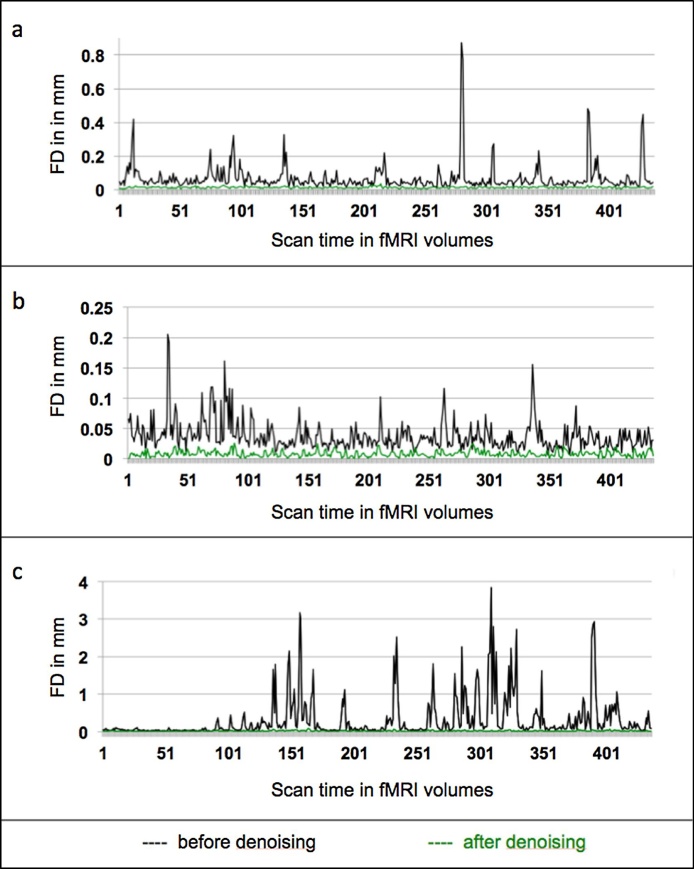
Average FD timecourse across participants for mean relative displacement before and after denoising in black and green respectively, is depicted in panel (a). Panel (b) shows an example of a low-motion scan before and after denoising, while panel (c) shows an example of a high-motion scan before and after denoising. No participants showed FD spikes >0.3 mm after denoising.

We assessed the success of our data cleaning procedure by computing motion parameters and framewise displacement in FSL on the denoised data. Details for head motion before and after data AROMA + FIX denoising were as follows: mean absolute displacement before (mean = 0.55 mm; SD = 0.07 mm); mean absolute displacement after (mean = 0.01 mm; SD = 0.004 mm); mean relative displacement before (mean = 0.85 mm; SD = 0.11 mm) and after (mean = 0.01; SD = 0.005) (average FD timecourse and examples of one low- and high-motion scan displayed in Fig. 1); number of volumes flagged using FD at 0.3 mm before (mean = 52.33, SD = 46.37); no spikes remained after denoising. We further quality-controlled our data using quality assessment scripts for functional data released by Roalf and colleagues (Kaufmann et al., 2017; Roalf et al., 2016) yielding estimates of each scan’s TSNR (see Kaufmann et al., 2017 for comparison). This was used as an additional measure to ensure comparable quality across all preprocessed scans (no scan exceeded > 3 SD from the group mean). Further, we observed that the TSNR increased noticeably from a mean of 65 (SD = 19.3) before denoising to a mean of 224 (SD = 42.24) after denoising. Histograms of motion-related measures before and after denoising are shown in Supplementary Fig. S1.

#### 2.5.4 fMRI analysis

The pre-processed and cleaned fMRI data were then subjected to an independent component analysis (ICA), as implemented in FSL’s MELODIC, using a multivariate exploratory linear decomposition in a temporal concatenation approach. Thirty group-level ICA components were extracted, among which three sensory and seven cognitive ICNs of interest were identified. ICA components were subsequently used to generate participant-specific versions of the spatial maps and associated timeseries using FSL’s dual regression approach (Filippini et al., 2009). Dual regression was used to identify, within each participant’s fMRI data, spatial maps and associated timecourses corresponding to the extracted ICA components. Dual regression was chosen as an analytic approach because it allows the generation of individual-subject maps of network membership, which can be then regressed against a parameter such as age, and also because it has been shown to have high test-retest reliability relative to seed-based techniques (Chen et al., 2015; Zuo et al., 2010; Zuo and Xing, 2014). The dual-regression procedure was carried out as follows. First, for each participant, the average group spatial maps of the ICA components were regressed into the participant’s four-dimensional (4D; space/time) dataset simultaneously. This resulted in a set of participant-specific time series, one per network. Then, these timeseries were regressed into the same 4D dataset, resulting a set of participant-specific spatial maps, one per network. This provided pairs of estimates which form a dual space and jointly best approximate the original group ICA maps.

Group associations with age within the sensory and cognitive ICNs of interest were then assessed using the respective ICN as a mask in FSL’s Randomise (5000 permutations (Winkler et al., 2014) and threshold-free cluster enhancement (Smith and Nichols, 2009) to estimate cluster activation) with p < 0.05 corrected for multiple comparisons (10 ICNs; p < 0.005); thus in this study we specifically examined intra-network FC, or “network integration”. IQ and handedness were entered as covariates of no interest. Age, IQ and handedness scores were converted to z-scores.

We hypothesized that networks showing associations with age are those that are most likely to be related with attention in this age range. To investigate associations with attention skills independent of age, this procedure was repeated for all ICNs that showed FC changes with age. Sustained, selective and executive attention skills were modelled separately due to their collinearity, with age, IQ and handedness included as covariates of no interest.

In order to ensure that significant findings were not due to head motion (Power et al., 2015), for all clusters that were significantly associated with age or attention scores, FC values were extracted from the cluster and correlated with participants’ motion in the scanner, quantified using FD, TSNR, mean absolute displacement (MAD), and mean relative displacement (MRD).

## 3 Results

### 3.1 Cognitive measures

Details for the attention measures were as follows: sustained attention (mean = 23.59; SD = 4.89; range 10–30); selective attention (mean = 14.15; SD = 2.73; range 6–18); executive attention (mean = 2.25; SD = 0.73; range 1–3). Histograms of attention score distribution are shown in Supplementary Fig. S2. Age significantly and positively correlated with sustained (r = 0.64, p < 0.000001), selective (r = 0.48, p = 0.0001) and executive attention (r =0.33, p = 0.01). Despite being standardized, IQ scores exhibited a weak negative correlation with age (r = −0.27, p = 0.037) but not attention (all p’s > 0.06). No significant correlations were found between age, attention, IQ or handedness and any of the motion parameters (all p’s > 0.06; see Supplementary Table S1).

### 3.2 Functional networks

**Fig. 2:**
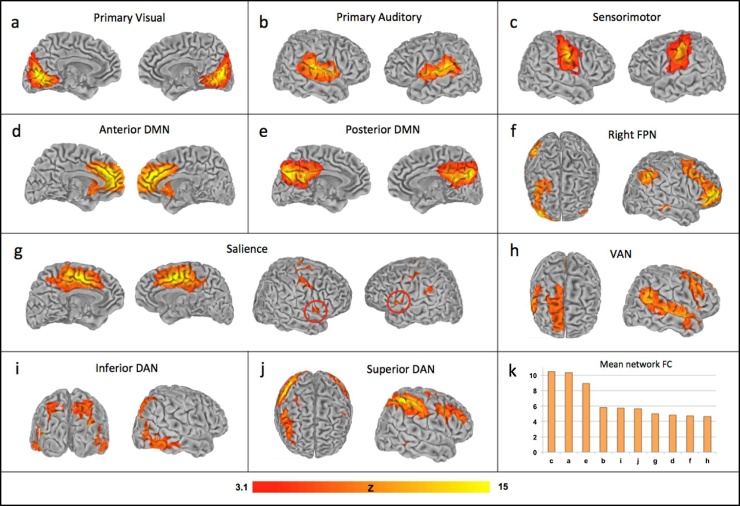
Sensory and cognitive networks of interest were extracted from the children’s cleaned passive viewing fMRI data via Independent Component Analysis (ICA). Network maps were thresholded at Z > 3.1 and projected onto surfaces in SUMA for visualization purposes. The following are shown in radiological convention: (a) primary visual network, (b) primary auditory network, (c) sensorimotor network, (d) anterior default mode network, (e) posterior default mode network, (f) right frontoparietal network, (g) salience network with the anterior insula circled in red, (h) ventral attention network, (i) inferior dorsal attention network, and (j) superior dorsal attention network. The average FC for each network is depicted in panel (k), ordered from highest to lowest.

Among the thirty components extracted via ICA, we identified several sensory and cognitive ICNs of interest based on spatial similarity to commonly observed ICNs (Beckmann and Smith, 2004; Damoiseaux et al., 2006) (Fig. 2): a primary visual network (a), a primary auditory network (b), a sensorimotor network (c), an anterior default mode network (d), a posterior default mode network (e), a right-lateralized frontoparietal network (f), a salience network (g), a right lateralized ventral attention network (h), an inferior dorsal attention network (i) and a superior dorsal attention network (j). Fig. 2k shows the average FC for each network, ordered from highest to lowest. We note that the sensorimotor, visual and posterior DMN ICNs showed relatively greater FC than salience, VAN, FPN and DAN ICNs.

### 3.3 Functional network integration associated with age

**Fig. 3:**
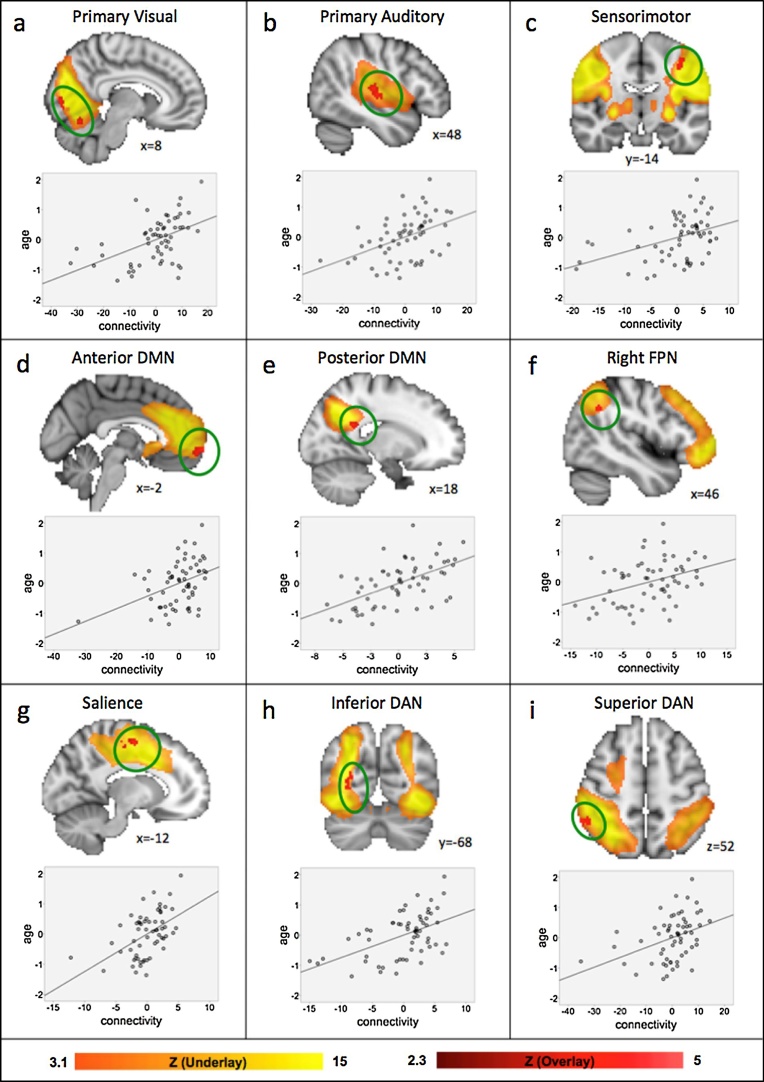
Associations with age in sensory and cognitive networks. Participant-specific versions of the spatial maps and associated timeseries were generated using FSL’s dual regression approach and associations with age within each ICN were assessed using FSL’s Randomise with p < 0.05 corrected for multiple comparisons while controlling for handedness and IQ. Positive associations with age were found in the (a) primary visual network, (b) primary auditory network, (c) sensorimotor network, (d) anterior default mode network, (e) posterior default mode network, (f) right frontoparietal network, (g) salience network, (h) inferior dorsal attention network, and (i) superior dorsal attention network. Significant associations with age are depicted in the red overlay; the yellow underlay depicts the respective network. Age is given in years (mean-centered); FC values are parameter estimates (β) in arbitrary units (mean-centered). DAN = dorsal attention network; DMN = default mode network; FP.

Age significantly and positively correlated with regional FC in all sensory and cognitive ICNs, with the exception of the VAN (Fig. 3 and Table 1). Specifically, regions showing stronger FC with older age were in primary sensorimotor areas (visual cortex V1–V2, primary auditory cortex, primary motor cortex) as well as in the cerebellum (lobules V–VI), and cognitive areas in the frontal and parietal lobes (anterior and posterior cingulate cortex, rostromedial PFC, inferior parietal lobule/angular gyrus and intraparietal sulcus). We note that network boundaries extend into white matter and clusters in the posterior DMN, the inferior DAN and the Salience network overlap with white matter. The relationship depicted in Fig. 3d includes a datapoint close to our pre-set criteria of 3 SD from the mean. A follow-up analysis revealed that this relationship, and all other age associations, remain significant with this participant removed. No significant negative associations were observed. Our sanity check to ensure associations were not driven by head motion showed that no significant correlations were found between FC values from clusters that were significantly associated with age, and any of the motion parameters (all p’s > 0.06; see Supplementary Table S2).

**Table 1:** Details for the associations with age in sensory and cognitive networks using FSL’s dual regression, permutation testing and threshold-free cluster enhancement. ACC = anterior cingulate cortex; BIL = bilateral; DAN = dorsal attention network; DMN = default mode network; FPN = frontoparietal network; IPL = inferior parietal lobule; L = left; Lat = laterality; PFC = prefrontal cortex; R = right.

Directionality	Network	Lat	Connectivity	Voxels	Z-Max	x	y	z
positive	Visual	R	Visual Cortex V1-V2	65	3.27	12	−84	2
		R	Cerebellar V, VI	61	2.95	6	−64	−18
positive	Auditory	R	Heschl's Gyrus H1	94	3.05	46	−22	10
positive	Sensorimotor	L	Motor Cortex M1	33	3.44	−38	−12	52
positive	anterior DMN	BIL	rostromedial PFC	31	3.01	−2	66	−6
positive	posterior DMN	R	Posterior Cingulate	50	4.14	18	−46	24
		L	Posterior Cingulate	44	3.71	−18	−46	24
positive	right FPN	R	IPL/Angular Gyrus	25	3.19	46	−52	34
positive	inferior DAN	R	Visual Cortex V1	60	3.82	32	−64	4
positive	superior DAN	R	Intraparietal Sulcus	23	3.11	56	−48	52
positive	Salience	BIL	dorsal ACC, SMA	30	3.58	−12	−8	56

### 3.4 Functional network integration associated with attention skills

**Fig. 4:**
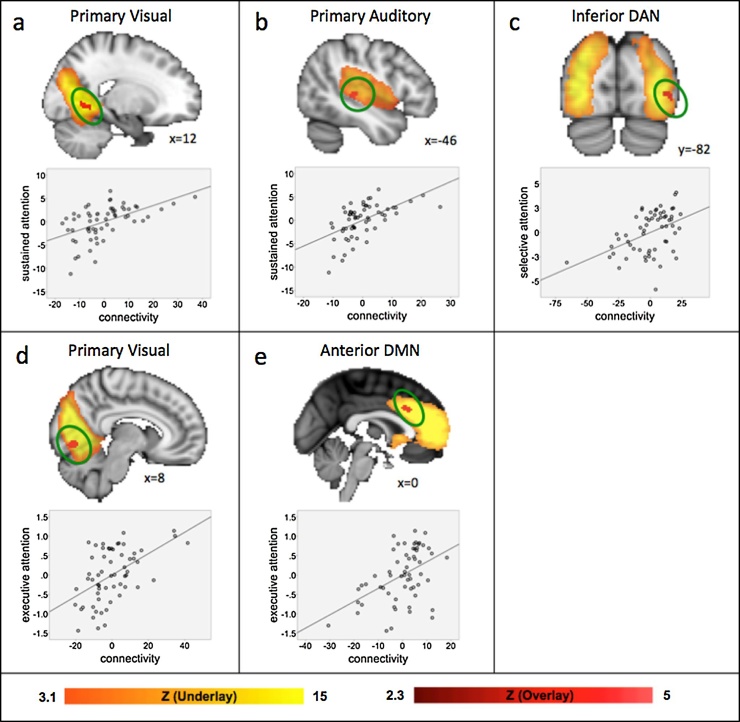
Associations with sustained, selective and executive attention in sensory and cognitive networks. Participant-specific version of the spatial maps and associated timeseries were generated using FSL’s dual regression approach and associations with attention within each ICN were assessed using FSL’s Randomise with p < 0.05 corrected for multiple comparisons while controlling for age in addition to handedness and IQ. Positive associations with sustained attention were found in the (a) primary visual network and the (b) primary auditory network; with selective attention in the (c) inferior dorsal attention network; and for executive attention in the (d) primary visual network, as well as the (e) anterior default mode network. Significant associations with attention are depicted in the red overlay; the yellow underlay depicts the respective network. Attention is given as score on the respective task (mean-centered); FC values are parameter estimates (β) in arbitrary units (mean-centered). DAN = dorsal attention network; DMN = default mode network.

Attention measures were significantly and positively correlated with FC in several distinct sensory and cognitive ICNs after controlling for the effects of age (Fig. 4 and Table 2). Specifically, greater FC in relation to greater sustained attention was found in primary visual and auditory cortices; greater FC in relation to greater selective attention was found in the inferior LOC of the dorsal attention network; and greater FC in relation to greater executive attention was found in an area spanning visual cortex 1, 2, 4, as well as in the dorsal ACC of the default mode network. Of note, the relationship depicted in Fig. 3c includes a datapoint close to our pre-set criteria of 3 SD from the mean. A follow-up analysis revealed that this relationship, and all other attention skill associations, remain significant when this participant is removed. No negative associations were observed and no significant correlations between FC values from clusters that were significantly associated with attention, or any of the motion parameters were found (all p’s > 0.25; see Supplementary Table S3).

**Table 2:** Details for the associations with attention measures that were found in sensory and cognitive networks after controlling for age. ACC = anterior cingulate cortex; BIL = bilateral; DAN = dorsal attention network; DMN = default mode network; L = left; Lat = laterality; LOC = lateral occipital cortex; R = right.

Correlated with	Network	Lat	Connectivity	Voxels	Z-Max	x	y	z
Sustained attention	Visual	R	Visual Cortex V1	53	4.32	22	−60	0
		R	Cerebellar V, VI	67	3.64	12	−68	−16
Sustained attention	Auditory	L	Heschl's Gyrus H1	53	4.81	−44	−30	0
Selective attention	inferior DAN	L	inferior LOC/MT+	44	4.46	−44	−80	−2
Executive attention	Visual	BIL	Visual Cortex V1-2, 4	347	3.78	20	−68	−6
		BIL	Cerebellar V	68	3.59	18	−54	−14
Executive attention	anterior DMN	BIL	dorsal ACC	35	3.97	45	74	52

## 4 Discussion

In this study, we investigated the association between age and FC within sensory and cognitive ICNs, and the association between FC in these ICNs and attention skills, in early childhood. Focusing on the age range of 4–7 years in order to capture the detailed changes that occur specifically during this period, we show in young girls that age is positively associated with increasing FC in most sensory and cognitive ICNs. We further found that, controlling for age, FC in the dorsal attention and default mode networks, as well as primary visual and auditory networks, was significantly associated with different components of attention. These findings fill a gap in our understanding of age-associations in functional networks between infancy and middle-to-late childhood, and suggest that the development of attention is related to functional integration of several distinct sensory and cognitive ICNs.

It has been suggested that cortical development proceeds in a hierarchical sequence from sensory to association cortex to cognitive regions (Gogtay et al., 2004; Shaw et al., 2008). As we find positive associations with age in primary visual and auditory, as well as sensorimotor networks in the current study, this suggests that sensory ICNs are indeed integrating in early childhood. Consistent with previous findings pointing to a linear maturation trajectory for sensory ICNs during the earliest developmental stages as well as late childhood to young adulthood, sensory ICNs were found to have increasing FC with age in a cross-sectional study on fetuses as young as 24–38 weeks gestational age (Thomason et al., 2013); therefore sensory ICNs may emerge as early as the beginning of the third trimester of gestation, the period of most rapid neuronal growth (Doria et al., 2010). They have been reported to already exist in rudimentary form in children born preterm (Doria et al., 2010), and are the easiest identifiable ICNs in newborns (Fransson et al., 2009). In a study contrasting newborns to 1- and 2-year-old children, Lin and colleagues examined sensorimotor and visual cortex regions-of-interest and detected significantly greater FC in the 2-year-olds vs. the 1-year-olds vs. the newborns (Lin et al., 2008). The only prior study to describe ICA-derived ICNs in an age range similar to our sample (de Bie et al., 2012) (5–8 years) found that sensory ICNs looked adult-like on visual inspection, but did not attempt to investigate associations with age – a gap that we have filled in this study. In older children and adolescents, two studies demonstrated reduced FC in sensory ICNs compared with young adults (Jolles et al., 2011; Kelly et al., 2009), indicating that sensory ICNs continue to change linearly with age until adulthood.

In contrast to the literature on sensory ICNs, current research suggests a non-linear maturational trajectory for cognitive ICNs. Thus, there may be qualitatively different developmental trajectories between sensory and cognitive ICNs. In infancy, cognitive ICNs exhibit greater FC with age (Gao et al., 2013). However, in children aged 11–13 years, FC in cognitive ICNs appears to be greater than in young adults (Jolles et al., 2011). The emergence of cognitive ICNs has been documented in primitive and incomplete versions of the DMN and DAN in two studies of newborns (Fransson et al., 2009; Gao et al., 2009). Specifically, Fransson et al. observed what they called a “proto-DMN” in the infant brain with strong FC between the precuneus/posterior cingulate cortex and the bilateral parietal cortex, but no significant FC between the posterior/medial aspects of the parietal cortex and the medial prefrontal cortex (Fransson et al., 2009). In the first two years of life, dramatic development of the DMN and DAN was observed, which went from large diffuse blobs surrounding the seed regions in newborns, to discernible architectures by the first year, with subtler changes from 1-year olds to 2-year olds (Gao et al., 2013). In the current paper, we demonstrate that regional or voxel-wise membership in cognitive ICNs is greater the older the children were, in line with the aforementioned work by de Bie et al. (2012) who, indicative of ongoing integration, observed that cognitive ICNs in young children visually appeared more fragmented than those of adults. Most developmental studies have found that cognitive ICNs develop during childhood and well into adolescence (Grayson and Fair, 2017). A more positive association between age and FC in a cognitive ICN, the DAN, was also found more recently in late-childhood/pre-adolescence relative to across-adolescence (Vinette and Bray, 2015), suggesting that sometime during adolescence DAN integration peaks and FC starts to decrease. Intriguingly, a negative relationship between age and FC in the DMN in children aged 6–10 years (Muetzel et al., 2016) has also been shown. Together, this work suggests network-specific “turning points” in the non-linear development of cognitive ICNs, which may differ from the trajectory of sensory ICNs. We note that the VAN, the only ICN in our analysis that did not exhibit a significant association with age, was recently shown to be detected with a probability of only ∼70% as compared to 77–91% for the other networks (Muetzel et al., 2016), therefore reliability may contribute to a non-significant association with age.

Broadly, our work shows evidence for functional integration across early childhood, in both cognitive and sensory/motor networks. While above we have discussed our findings in relation to networks in infancy and childhood, it is also worth situating our work within the context of lifespan ICN changes (for more thorough reviews see (Grayson and Fair, 2017; Zuo et al., 2017)). A study of individuals from 7 to 85 years found less within-network connectivity and more between-network connectivity (Betzel et al., 2014) with less modularity the older participants were. This study and others (Wang et al., 2012) describe non-linear inverted-U FC patterns in some networks, though specifics diverge between studies (e.g. fronto-parietal in Wang et al. (2012) and salience/ventral attention in Betzel et al. (2014)). Taking a graph theoretical approach to examine lifespan network properties, Cao et al. (2014) found decreased modularity with age, despite relative stability in global efficiency. These findings are similar to Chan et al. (2014) who describe less system segregation, i.e. more between-system FC accompanied by less within-system FC. Overall, despite differences in the literature regarding which networks are most affected, there is relative consistency in reports of a decline in within-network FC across healthy aging (Tsang et al., 2017). Despite apparent symmetry to greater integration described here in early childhood, we (and others (Craik and Bialystok, 2006)) caution against the interpretation that aging is ‘undoing’ the maturation that occurs across childhood. The fact that we did not observe negative brain-behavior associations is in line with previous research suggesting linear maturation within ICNs in the age range studied here. Decreasing FC with age has however been shown in older children (e.g. Muetzel et al., 2016 studied children aged 6–10) or using centrality metrics (Long et al., 2017).

Importantly, we were able to link these integrative processes to individual variability in attention skills. As in our previous work (Rohr et al., 2017), we demonstrate that FC in the DAN is associated with selective attention skills in early childhood. Unlike in our previous seed-based analysis, where we observed enhanced FC between the intraparietal sulcus and the putative human frontal eye fields, here a higher-order visual region encompassing area MT+ and lateral occipital cortex showed the greatest age-association in voxel-wise ICN membership. This finding may have resulted from our use of ICA in this study, which allows for the examination of the whole network rather than a priori seed regions-of-interest. Of note, no other ICN showed this relationship, highlighting the DAN’s unique role in selective attention. Work in adults has shown an enhancement of DAN FC during selective attention (Szczepanski et al., 2013). Our finding that DAN FC is associated with inter-individual variability in selective attention skills suggests an experience-dependent refinement of this network in children with better attention skills: Gabard-Durnam et al. (2016) recently produced evidence that the neural activity underlying looking at emotional faces can shape intrinsic FC years later in life. Therefore, greater DAN FC might reflect an enhanced use of the ICN by children who have better selective attention abilities.

We further found, somewhat surprisingly, that stronger executive attention was related to stronger FC in the DMN, specifically the dorsal ACC. The dorsal ACC has been implicated in attention control (Aarts and Roelofs, 2011) and several associated aspects of executive functioning, such as decision making (Bush et al., 2002), and emotion regulation (Giuliani et al., 2011). Further, the DMN is sometimes thought of as “the brain’s autopilot”. In this regard, it is noteworthy that our task was akin to a Wisconsin Card Sorting Test and therefore assesses set-shifting and working memory in addition to executive attention. The dorsal ACC also has a hub function in the salience network (Seeley et al., 2007). The salience network is a causal mediator between the DMN and the DAN, meaning it can influence switching between attentional states (Uddin et al., 2011). Sustained attention, for instance, has been found to alternately rely on the DAN and DMN, when operating in effortful or relatively effortless modes (Esterman et al., 2014). Lastly, some differences exist in regard to how networks, or network components, are labeled, and a medial-to-lateral prefrontal network component of the spatial extent we observed here is sometimes labeled “Executive Control” network (see e.g. Muetzel et al., 2016), which again highlights the component’s relevance in executive attention.

Lastly, our finding that better sustained and executive attention skills in older children was related to greater FC in different parts of the primary visual ICN is broadly in line with previous work in both typically developing children and in children with attention deficit hyperactivity disorder (ADHD). In a whole-brain, seed-based analysis on data from infants and toddlers, Eggebrecht and colleagues (2017) found the strongest positive associations between FC and joint attention in the visual cortex. Visual and auditory processing has been found to be altered in children with ADHD (Hale et al., 2014; Kibby et al., 2015), and alterations in brain structure and function often include alterations in visual (Hale et al., 2014) and auditory networks (Heinrichs-Graham et al., 2014; Serrallach et al., 2016), alongside alteration in the DMN (Hale et al., 2014) and the DAN (Friedman and Rapoport, 2015). In addition, the size and synchronization of the auditory cortex has also been linked to attention skills in children (Seither-Preisler et al., 2014), providing further indication for the auditory cortex’ role in attention. In adults, FC between the IPS and visual regions is increased during sustained visual attention (Greenberg et al., 2012; Lauritzen et al., 2009). While some of the findings of the visual and auditory cortex in relation to attention may be influenced by the children perceiving visual and auditory stimuli via watching a TV show, it has also recently been shown that a distributed network referred to as the sustained attention network (SAN), mediates inter-individual variability in sustained attention skills (Rosenberg et al., 2016). Therefore it is conceivable that the roles of regions within ICNs that lie outside the networks typically associated with attention may have previously been underestimated. Our results thus underscore the importance of whole-brain approaches when assessing the neural correlates of complex cognitive skills such as sustained attention.

Despite the fact that children in this study were engaged in passive viewing of video clips during functional data acquisition, rather than at rest or sleep, the ICN patterns we observed are largely consistent with those observed in younger (Fransson et al., 2009; Gao et al., 2013) and older children and adults (Jolles et al., 2011). It has been previously shown that networks are largely similar, though not identical, during free-viewing and rest (Bray et al., 2015; Emerson et al., 2015; Vanderwal et al., 2015). Showing videos increases young children’s ability to stay still during a scan (Raschle et al., 2009), and may be especially useful for studies in children with neurodevelopmental disorders, many of whom evidence attention difficulties in addition to challenges staying still for MRI acquisitions (Atkinson and Braddick, 2011; Bray et al., 2011a; Bray et al., 2011b; Bray et al., 2013; Keehn et al., 2013; Rosenberg et al., 2016; von Rhein et al., 2015). It was further recently shown that individual differences in FC are enhanced during passive viewing, thus facilitating their detection not only through reduced motion but also through the synchronization of hemodynamic fluctuations in large areas of the cortex (Vanderwal et al., 2017). As a limitation, however, watching a TV show may influence relationships between FC and age or attention skills: differences in FC with age or attention could relate to different degrees of engagement with the show. In the future, this possibility could be tested using ratings of enjoyment (i.e. emotional response) and immersion in the TV show (Gaebler et al., 2014; Rohr et al., 2016) as well as eye-tracking (Cox et al., 2006) or heart rate and skin conductance (Mauss and Robinson, 2009; Rohr et al., 2016; Yeo et al., 2017).

In this study, we used a developmentally appropriate measure of selective, sustained and executive attention skills (Breckenridge et al., 2013), and as expected, found significant effects of age across the age range studied. However, the ecological validity of such assessments is somewhat limited, and a more nuanced view of children’s attentional abilities is emerging (Ristic and Enns, 2015), namely one that takes situational context into account. For example, developmental differences in visual short term memory capacity that are present when a child is asked to remember a series of letters is no longer present when the target stimuli are pictures (Sørensen and Kyllingsbaek, 2012). When presented with emotional and non-emotional targets in a selective attention task, both adults and 9–12 year old children were faster at identifying the ‘fearful’ targets and between-group differences were non-significant (Waters et al., 2004). Children of different ages, and hence communication abilities, look differently at social scenarios; specifically, younger children spent more time looking at eyes relative to the mouth (Frank et al., 2011), demonstrating that children prioritize their attention to stimuli differently depending on their own personal context. This literature suggests that our results may be context-specific and more work is needed to understand how the skills measured here relate to real-world abilities.

The current study has several distinct strengths. By focusing on the relatively narrow age range of 4–7 years, we are better able to capture the detailed changes that occur specifically during this period. Other strengths include the use of a multi-component measure of attention, training in an MRI simulator to acquaint children with the scanning environment, a sufficiently long scan time to obtain reliable estimates of FC, and a method well-suited to investigate multiple ICNs at once. In addition, both the denoising and dual regression methods employed here have been shown to increase reproducibility of spatial maps representing ICNs, which is particularly relevant for a neurodevelopmental study (Chen et al., 2015; Pruim et al., 2015; Zuo and Xing, 2014). The study also has several limitations. First, the data was collected as part of an ongoing study of genetic disorders affecting girls, and as a result boys were not included. It has been shown that there are no significant sex differences in attention skills in this age range (Breckenridge et al., 2013); therefore, we expect results to generalize, but this should be tested in future work. Second, while associations with age are suggestive of developmental effects, longitudinal data is necessary to confirm within-subject maturation and the relationship between FC changes and changes in attention measures. Future studies could make use of longitudinal cohort data as it becomes more readily available online through various projects around the globe. Third, clusters that we observed in the posterior DMN, the inferior DAN and the Salience network are primarily in the white matter. This may be indicative of imperfect registration in some brain areas. ICA networks specifically, however, also tend to cover large amounts of white matter, as they span across several grey matter areas. Arbitrary thresholding of network masks (e.g. a z-value of 3.1 vs. 2.3) can make a difference where the bounds are, meaning a cluster in the white matter could be the result of a cluster peak in a portion of grey matter that was cut off in the masking process. Fourth, between-network effects were not assessed due to limited power, but will be important to assess in future work. Lastly, while handedness was included as a covariate in our analyses, some effects associated with it may not be continuous along a linear spectrum.

The characterization of the emergence of ICNs in children has been of immense interest to researchers focusing on typical and atypical developmental trajectories. In this work we show that sensory, as well as cognitive ICNs show functional integration across the period of early childhood. We reproduced findings that ICNs are more diffuse at the earlier stages of childhood and extend the research literature by demonstrating that the functional integration that occurs in cognitive ICNs later in childhood and partially throughout adolescence, is well underway in early childhood. Notably, previous work has shown that selective, sustained and executive attention are essentially mature by mid-adolescence (Zhan et al., 2011). Therefore, increasing regional membership in cognitive ICNs in early childhood might reflect network mechanisms that support the acquisition of attention, and other cognitive skills, which become attenuated once these skills are acquired and consolidated. This latter process may be associated with a decline in FC into adulthood. In addition, our data confirms that sensory ICNs undergo continuous refinement in children aged 4–7 years. Importantly, we were able to demonstrate that greater sustained, selective and executive attention skills are accompanied by functional network integration in distinct cognitive and sensory ICNs during early childhood. Given the ubiquity of attention difficulties in children with neurodevelopmental disorders, this further highlights the tremendous potential for early childhood therapies to curb atypical developmental trajectories.

## Conflict of interest

None.
