# Supplementary material for: Functional network integration and attention skills in young children
Source: Dev Cogn Neurosci. 2018 Mar 20;30:200–11. doi: 10.1016/j.dcn.2018.03.007 (PMC6969078; doi:10.1016/j.dcn.2018.03.007)
Supplement: Supplementary file 1 [file mmc1.docx]

**SUPPLEMENTARY MATERIALS**

**
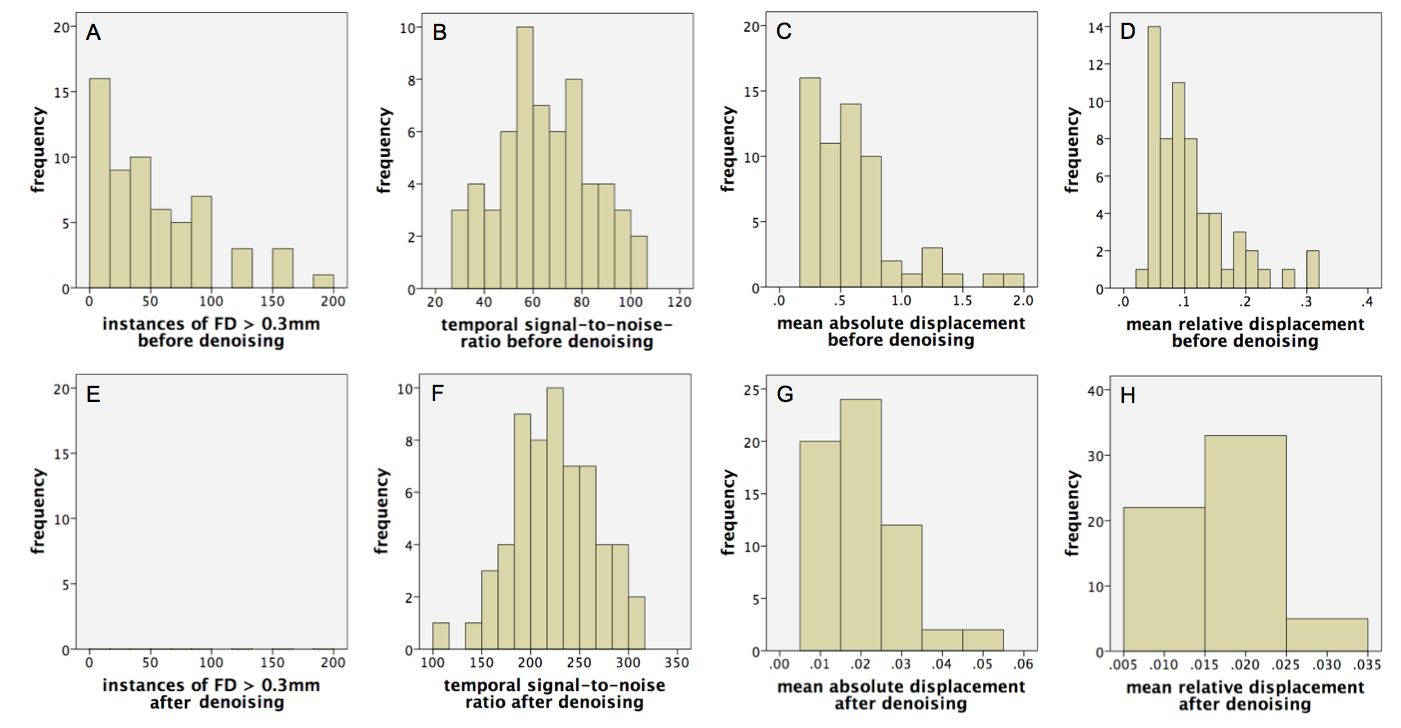
**

**Supplementary Figure S1. Distribution of motion assessment parameters.** FD is given in number of volumes flagged (instances of framewise displacement > 0.3mm), temporal signal-to-noise ratio is given in arbitrary units, displacements are given in mm.

**
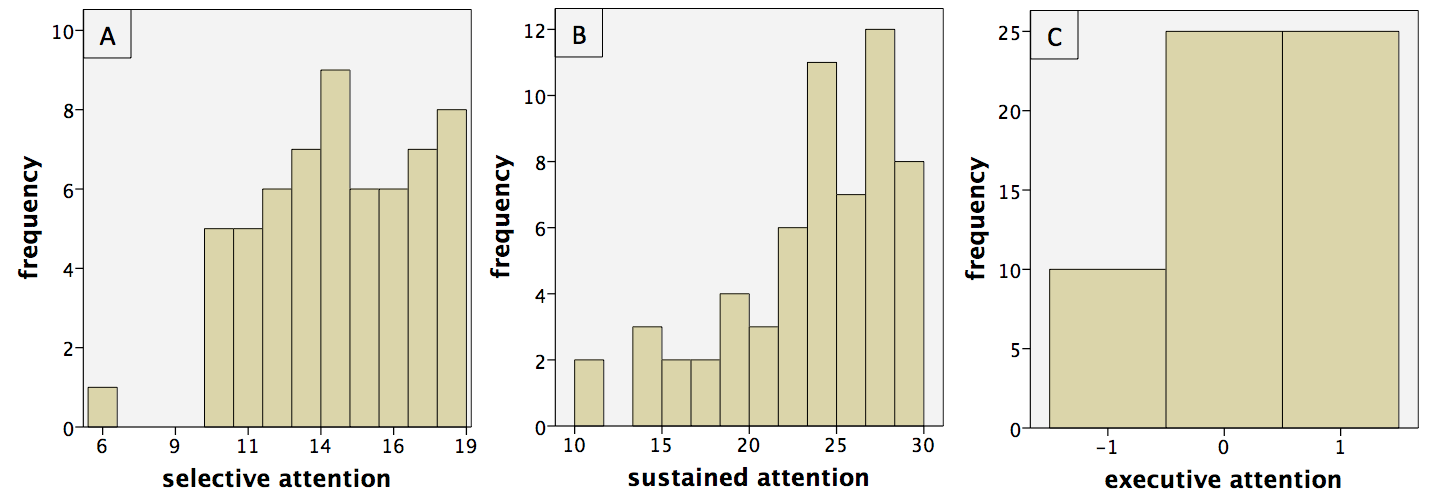
**

**Supplementary Figure S2. Distribution of attention scores.**

| *Correlation with* |  | FD | TSNR 1 | MAD 1 | MRD 1 | TSNR 2 | MAD 2 | MRD 2 |
| --- | --- | --- | --- | --- | --- | --- | --- | --- |
| age | r | 0.00 | -0.04 | 0.15 | 0.08 | -0.15 | 0.21 | 0.01 |
|  | p | 0.98 | 0.75 | 0.25 | 0.56 | 0.25 | 0.11 | 0.41 |
| selective attention | r | -0.09 | 0.01 | 0.04 | -0.03 | -0.07 | 0.04 | 0.06 |
|  | p | 0.48 | 0.93 | 0.77 | 0.80 | 0.58 | 0.75 | 0.67 |
| sustained attention | r | 0.12 | -0.11 | 0.17 | 0.16 | -0.21 | 0.18 | 0.13 |
|  | p | 0.38 | 0.42 | 0.21 | 0.22 | 0.10 | 0.18 | 0.34 |
| executive attention | r | -0.01 | -0.05 | -0.06 | 0.06 | -0.13 | 0.01 | 0.05 |
|  | p | 0.96 | 0.70 | 0.63 | 0.67 | 0.32 | 0.93 | 0.72 |
| IQ | r | 0.17 | -0.19 | 0.07 | 0.13 | -0.25 | 0.16 | 0.20 |
|  | p | 0.18 | 0.15 | 0.58 | 0.31 | 0.06 | 0.22 | 0.13 |
| handedness | r | -0.02 | 0.12 | 0.04 | 0.05 | 0.12 | -0.18 | -0.24 |
|  | p | 0.88 | 0.38 | 0.75 | 0.71 | 0.38 | 0.18 | 0.08 |

**Supplementary Table S1. Details for the associations between age, attention measures, IQ, handedness and motion assessments in the final sample.** No significant correlations were observed. FD=framewise displacement; MAD=mean absolute displacement; MRD=mean relative displacement; TSNR=temporal-signal-to-noise ratio.

| *Correlation with age-related FC* |  | FD | TSNR 1 | MAD 1 | MRD 1 | TSNR 2 | MAD 2 | MRD 2 |
| --- | --- | --- | --- | --- | --- | --- | --- | --- |
| Visual | r | -0.03 | 0.02 | -0.06 | -0.01 | -0.04 | 0.06 | 0.08 |
|  | p | 0.84 | 0.90 | 0.64 | 0.94 | 0.79 | 0.63 | 0.53 |
| Auditory | r | -0.06 | -0.12 | 0.02 | -0.04 | 0.14 | 0.08 | -0.03 |
|  | p | 0.63 | 0.38 | 0.91 | 0.76 | 0.31 | 0.56 | 0.80 |
| Sensorimotor | r | -0.17 | 0.09 | -0.06 | -0.12 | 0.08 | 0.03 | -0.03 |
|  | p | 0.19 | 0.52 | 0.68 | 0.39 | 0.56 | 0.85 | 0.80 |
| anterior DMN | r | 0.04 | 0.03 | 0.04 | 0.12 | -0.08 | -0.02 | -0.05 |
|  | p | 0.76 | 0.81 | 0.78 | 0.37 | 0.53 | 0.86 | 0.72 |
| posterior DMN | r | -0.01 | -0.20 | 0.15 | 0.04 | -0.06 | 0.00 | 0.01 |
|  | p | 0.93 | 0.14 | 0.25 | 0.75 | 0.68 | 0.97 | 0.92 |
| right FPN | r | -0.10 | -0.10 | -0.15 | -0.06 | 0.15 | 0.08 | 0.16 |
|  | p | 0.47 | 0.46 | 0.27 | 0.68 | 0.25 | 0.56 | 0.23 |
| inferior DAN | r | 0.15 | -0.05 | 0.25 | 0.24 | -0.19 | 0.05 | -0.01 |
|  | p | 0.27 | 0.71 | 0.06 | 0.08 | 0.16 | 0.72 | 0.97 |
| superior DAN | r | -0.02 | 0.00 | -0.03 | 0.02 | -0.01 | 0.07 | 0.06 |
|  | p | 0.89 | 0.99 | 0.82 | 0.87 | 0.94 | 0.63 | 0.63 |
| Salience | r | 0.10 | 0.04 | -0.06 | 0.10 | -0.11 | -0.12 | -0.07 |
|  | p | 0.46 | 0.76 | 0.65 | 0.44 | 0.41 | 0.35 | 0.60 |

**Supplementary Table S2. Details for the associations between age-related FC values and head motion parameters to assess the presence of any remaining impacts.** Correlations were controlled for handedness and IQ as in the randomize model. No significant correlations were observed. FD=framewise displacement; MAD=mean absolute displacement; MRD=mean relative displacement; TSNR=temporal-signal-to-noise ratio.

| *Correlation with attention-related FC* |  | FD | TSNR 1 | MAD 1 | MRD 1 | TSNR 2 | MAD 2 | MRD 2 |
| --- | --- | --- | --- | --- | --- | --- | --- | --- |
| Visual (sustained attention) | r | 0.08 | -0.08 | 0.16 | 0.11 | -0.12 | -0.01 | -0.04 |
|  | p | 0.57 | 0.55 | 0.25 | 0.43 | 0.37 | 0.97 | 0.80 |
| Auditory (sustained attention) | r | 0.10 | 0.01 | 0.04 | 0.10 | -0.12 | -0.08 | -0.05 |
|  | p | 0.44 | 0.95 | 0.79 | 0.47 | 0.39 | 0.54 | 0.69 |
| inferior DAN (selective attention) | r | -0.10 | 0.03 | -0.05 | -0.02 | 0.10 | -0.07 | -0.02 |
|  | p | 0.48 | 0.85 | 0.72 | 0.90 | 0.46 | 0.63 | 0.87 |
| Visual (executive attention) | r | -0.11 | 0.04 | -0.11 | -0.10 | 0.02 | -0.11 | -0.12 |
|  | p | 0.40 | 0.76 | 0.40 | 0.45 | 0.90 | 0.44 | 0.38 |
| anterior DMN (executive attention) | r | 0.06 | -0.12 | 0.04 | 0.13 | -0.14 | 0.00 | 0.11 |
|  | p | 0.67 | 0.37 | 0.75 | 0.33 | 0.31 | 0.99 | 0.42 |

**Supplementary Table S3. Details for the associations between attention-related FC values and head motion parameters to assess the presence of any remaining impacts.** Correlations were controlled for handedness and IQ as in the randomize model. No significant correlations were observed. FD=framewise displacement; MAD=mean absolute displacement; MRD=mean relative displacement; TSNR=temporal-signal-to-noise ratio.
